# The Role of Genetics in the Development and Pharmacotherapy of Depression and Its Impact on Drug Discovery

**DOI:** 10.3390/ijms24032946

**Published:** 2023-02-02

**Authors:** Agata Zięba, Dariusz Matosiuk, Agnieszka A. Kaczor

**Affiliations:** 1 Department of Synthesis and Chemical Technology of Pharmaceutical Substances with Computer Modeling Laboratory, Faculty of Pharmacy, Medical University of Lublin, 4A Chodźki St., PL-20093 Lublin, Poland; 2 School of Pharmacy, University of Eastern Finland, Yliopistonranta 1, P.O. Box 1627, FI-70211 Kuopio, Finland

**Keywords:** gene polymorphism, drug resistance, GPCRs, schizophrenia, depression, computer-aided drug discovery

## Abstract

Complex disorders, such as depression, remain a mystery for scientists. Although genetic factors are considered important for the prediction of one’s vulnerability, it is hard to estimate the exact risk for a patient to develop depression, based only on one category of vulnerability criteria. Genetic factors also regulate drug metabolism, and when they are identified in a specific combination, may result in increased drug resistance. A proper understanding of the genetic basis of depression assists in the development of novel promising medications and effective disorder management schemes. This review aims to analyze the recent literature focusing on the correlation between specific genes and the occurrence of depression. Moreover, certain aspects targeting a high drug resistance identified among patients suffering from major depressive disorder were highlighted in this manuscript. An expected direction of future drug discovery campaigns was also discussed.

## 1. Introduction

Technological development has significantly influenced the field of science and led to the creation of numerous modern tools, which have enabled the establishment of links between factors previously considered unimportant. Therefore, it is now possible to notice the subtle, but significant nuances and with their help optimize the therapeutical strategy. This provides us with valuable information on the pathomechanism of certain diseases and supports all previous findings on their indeed complex nature. Modern genomic approaches enable researchers to explore variations in the genetic material in a hypothesis-free manner, with no need to preselect genes of interest. Thanks to that, it is possible to find novel genes associated with a certain disorder. On the other hand, the large quantities of data available in this field make dreams of the development of one effective medication capable of equally diminishing symptoms in all patients fade away. The more substances are being marketed and the more data on their effectiveness are being collected it is becoming more recognizable that not every implementation is a great success, and despite promising results obtained in pre-clinical and clinical trials, there could be a group of patients who do not respond well to the treatment. Although this phenomenon can be identified in numerous conditions, currently the field of mental health diseases is becoming a topic of serious global concern. 

**Figure 1:**
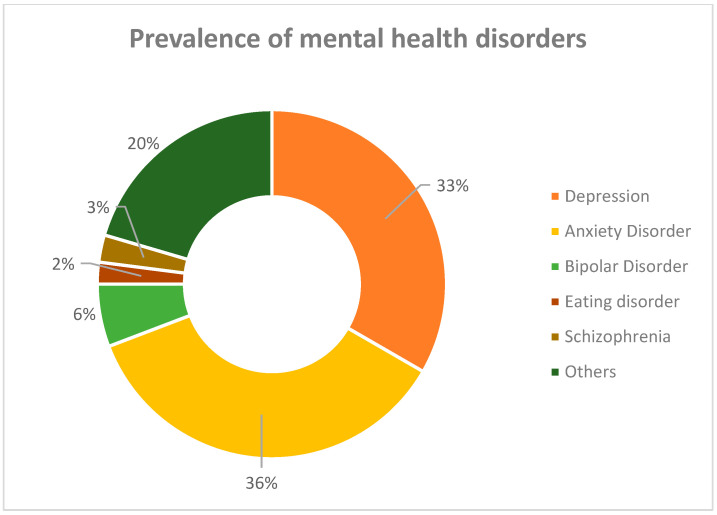
Epidemiological data for the prevalence of mental disorders in 2017 [1].

According to a report produced by the Institute for Health Metrics and Evaluation in 2017, around 792 million people were diagnosed with a mental health disorder worldwide. Figure 1 presents a graphical interpretation of the prevalence data of several disorders distinguished in surveys for both sexes. It can be easily noticed that both depression and anxiety disorders comprise more than half of all identified conditions [1].

Up till that point, statistically, 1 in 10 people suffered from such a disorder. Currently, this number could be significantly higher. This is supported by, e.g., the scientific brief published in 2022 by the World Health Organization, which revealed that the Coronavirus disease 2019 (COVID-19) pandemic harmed the mental health of citizens, and current estimates of the prevalence of mental disorders were expected to increase by 25% after the first year of the pandemic [2]. These data emphasize the urgent need to revise the current state of knowledge on the pathomechanism of mental disorders and the effectiveness of currently applied therapeutical strategies. Subsequent to the fact that the prevalence of depression and anxiety disorder significantly exceeds the incidence of other classified mental disorders, and the quantity of data available for each of these conditions are enormous, this article will focus on only one of the abovementioned diseases.

**Figure 2:**
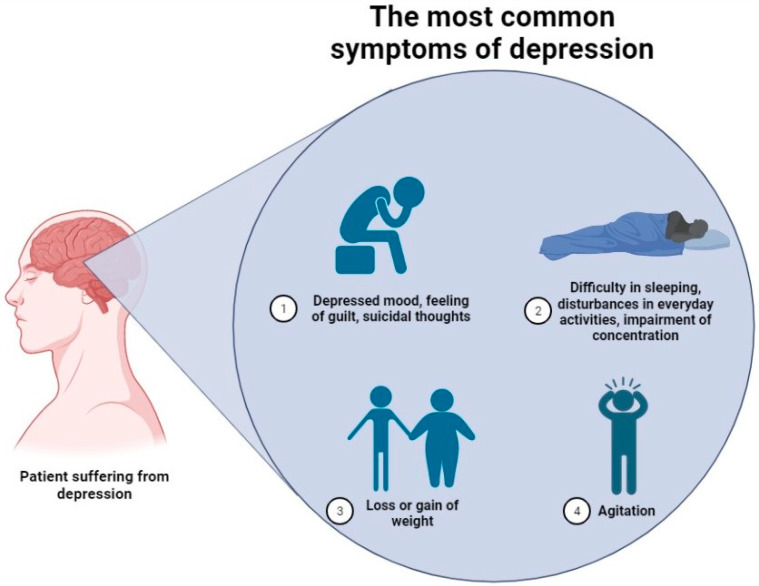
The most common symptoms of depression are included in the Hamiltonian Depression Rating Scale (HDRS) [5,6].

Depression is a complex psychiatric abnormality, where numerous harmful factors can contribute to the development of the condition. Despite the high social significance of the disease confirmed by, e.g., statistical data, no clear concept explaining the clinical picture of the disorder exists. Over the years, several hypotheses have been introduced to explain it and these include “monoamine”, and “cytokine” models or the altered neuronal plasticity phenomenon [3,4]. However, none of these is capable of explaining the exact cascade of reactions leading to the occurrence of characteristic symptoms. Figure 2 gathers the most common symptoms of depression included within the Hamiltonian Depression Rating Scale (HDRS), a widely used clinical depression assessment scale [5]. Clinical symptoms of the disease consist of a set of reactions, which can be easily misinterpreted; thus, the exact number of people suffering from the disorder can be significantly different. Therefore, from our perspective, both the abovementioned factors, along with the fact that numerous marketed antidepressants do not cure the disease but only reverse its symptoms, became an incentive to thoughtfully evaluate the available data. 

It is worth emphasizing that recently, a great number of articles have been published in this field, meticulously evaluating the physiological changes occurring in human cells and summarizing current pharmacological advances and directions. However, significantly fewer documents focus on the role of genetics in the disease. Therefore, to exploit this information gap, we decided to create a document summarizing the impact of genes on the clinical picture of the disease, with a special emphasis on its links to the effectiveness of pharmacotherapy. Moreover, within this manuscript, modern computational approaches will be discussed, since they constitute a valuable tool for the analysis of such data. Finally, based on the collected information, we will try to determine the importance of these findings for the mitigation of disease symptoms in treatment-resistant patients suffering from major depressive disorder (MDD).

## 2. Hereditary Picture of Depression

Heritability estimates for different forms of depression range from 30 to 50%, depending on the data source [7]. Typical hereditary occurs when it is possible to name specific alterations in genes or chromosomes which lead to the development of a certain condition. These changes are transferred from the parent to the child [8]. In the case of depression and other complex diseases, certain genetic alterations play only a partial role in the development of the condition. The remaining factors, involved in the generation of depression-like symptoms are considered non-genetic and include, e.g., environmental factors, administered therapeutic agents, and co-existing disorders [9].

Heretofore, numerous studies, which focused on the determination of the disease risk for different forms of this disorder, in numerous groups of related patients, were published. One way to examine the correlation between genetics and the epidemiology of a certain disorder is to perform family or adoption studies, based on data derived from large national registers [10]. A great extent of the studies referring to depression involves the presence of both monozygotic and dizygotic twins. Identical twins (monozygotic) share essentially identical genetic material; therefore, possible phenotypic alterations result from the presence of external, e.g., environmental factors. On the other hand, dizygotic (fraternal) twins carry significantly different genetic material. The classical organization of a twin study involves the examination of certain factors in representatives of both above-mentioned groups. If there is a high similarity identified in monozygotic twins, rather than in dizygotic twins, this supports the high relevance of genes in the development of certain feature or disorder [11].

Some of the most distant works in this field come from the 20th century and have estimated the ratio for an increased risk of development of major depression in first-degree relatives of patients for 2.84 [12]. Moreover, in the early 2000s, an interesting work examining the heritability of depressive symptoms in children was published by Happonen et al. The introductory part of the article contains a critical analysis of the former establishments in this field. It emphasizes possible drawbacks of each of the previously conducted studies such as inconsistency in information obtained from certain informants and lack of definitive criteria for valid measures. The main part of the article focuses on the heritability of depressive symptoms among relatives of various sexes. The presented results were based on the large sample of 1366 participants, comprising 11- and 12-year-old twin pairs. Although the estimates of additive genetic effects were significant for groups of boys and girls, the correlation of teacher and parent ratings was poor, and the bivariate model-fitting procedure showed no significant correlation in genetic and shared environmental effects between these two ratings. For children at such a young age, sex-differentiating effects were only noticed in teacher ratings, where genetics seemed to have a greater impact on girls than boys [13]. On the other hand, Jansson et al. investigated the importance of both genetics and environmental factors on the development of depression-like symptoms in a group of 959 elderly twin pairs. Observation of this group of patients, with a mean age of 72, showed a moderate heritability of depressive symptoms in elderly people. Women were more likely to develop these effects than men, although obtained differences for disease heritability were considered not statistically significant [14]. In addition, results from the Swedish national twin study of lifetime major depression, conducted on a large group exceeding 42,000 candidates of both dizygotic and monozygotic twins showed a moderate heritability profile of the disorder. Researchers concluded that one’s genetic risk factor for the development of depression was estimated at 42 and 29 percent in women and men, respectively [15]. Moreover, another large population study published in 2015 by Fernandez- Pujals et al. supported previous findings on the moderate influence of genetics on disorder development. Various genetic vulnerabilities to developing the major depressive disorder in females and males can be expressed as the correlation rate of 0.75 for both sexes, and 0.43; 0.99 for males and females, respectively. This particular study also examined the correlation between an individual’s sex and the disease onset time. In this second case, the genetic correlation between males and females with MDD was 0.85, and 0.66 to 0.98 for men and women [16].

Many of the heredity analyses unequivocally highlighted the significantly higher incidence of depression in women than in men. Unfortunately, current data are insufficient to provide a straightforward answer explaining the grounds for such a state. A trial conducted on a group of female cynomolgus monkeys emphasized the role of hormonal changes, especially estrogens, in the development of the symptoms of the disorder [17]. This seems to be supported by the fact that hormone replacement therapy is considered effective in the prevention of postmenopausal depression in women [18]. On the other hand, androgens could exhibit a protective effect in male hippocampal neurons [19]. Another possible group of factors enumerated while explaining this discrepancy refers to social grounds. According to some researchers, depression is less frequently identified in male subjects, because the ethos of hegemonic masculinity makes them ignore the symptoms of the disorder and reject offered help [20]. Heritability estimates are usually hard to compare between populations since they depend on the studied population, ascertainment, utilized diagnostic instruments, or even study conceptualization. Constant advances in research may influence the update of diagnostic criteria, which can also affect the abovementioned parameter [21,22]. Even though, the presented data show only a certain part of heritability studies, referring to large population data, these pieces of evidence show that depression is substantially heritable. Moreover, a disorder of this kind is more prevalent in women than men.

## 3. Studies on the Contribution of Certain Genes

Even though there is strong evidence confirming the high heritability of depression, it is impossible to name one genetic alteration leading to the development of this condition. Therefore, the current pathophysiological concept distinguishing numerous genes potentially involved in the pathomechanism of depression enabled us to notice differences observed in the clinical picture and treatment outcomes in representatives of both sexes [23]. One of the first attempts for the identification of gene candidates, potentially contributing to the development of affective disorders, was made in the late 70s. Then, the frequencies of certain serum groups (Hp, Gc) and red cell enzyme types (PGM, ESD, 6-PGD) were evaluated in a group of 195 patients suffering from depressive psychosis, non-psychotic reactive depression, or a certain group of unclassified patients [24]. The above-mentioned study discovered that there was no correlation between the identification of polymorphisms of Gc and 6-PGD systems in patients suffering from examined affective disorders. However, the Hp^2^ gene was increased in reactive patients, who were not classifiable, while the PGM_1_^1^ variant was more frequently observed in bipolar patients and ES D^1^ in reactive patients. 

For a long time, the vast majority of published studies targeting the impact of genetics on the clinical picture of depression focused on some functional polymorphisms in the loci encoding proteins associated with two pathways of two neurotransmitters—dopamine, and serotonin. The rationale behind this refers to the fact that both these chemicals are involved in the regulation of mood and behavior responses in human and laboratory animals [25]. Therefore, it was believed that collecting large quantities of data on these proteins will assist in the discovery of a potent antidepressant, while extensive knowledge of their polymorphisms could help to explain, e.g., the reasons behind treatment resistance.

The serotonin 1A receptor (5-HT_1A_) is encoded by the intronless HTR1A gene, located in chromosome 5. The most widely investigated variants of these genes include: C(−1019)G (rs6295); Gly272Asp (rs1800042); Ile28Val (rs1799921); Arg219Leu (rs1800044); and Gly22Ser (rs1799920) [25].

Studies using conditional knockout of a silencer Freud-1 (encoded by the Cc2d1a gene) of the 5-HT_1A_ receptor gene in serotoninergic neurons lead to the elevation of anxiety levels in laboratory animals. Moreover, this modification resulted in hypothermia and a reduction in serotonin (5-HT) levels. Interestingly, the knockdown of Cc2d1a/Freud-1 in the hippocampus of animal models did not affect the 5-HT_1A_ and D_2_ receptors’ expressions [25]. Another study targeted the importance of a polymorphism in the -1019C/G promoter region (rs6295) of the 5-HT_1A_ receptor gene. The homozygous G-allele identified in this polymorphism was associated in studied subjects with a worse response to a medication. Thus, the presence of this polymorphism leads to the loss of the control of gene expression by the nuclear-deformed epidermal autoregulatory factor-1-related inhibitory factor (NUDR), the elevation of the 5-HT_1A_ receptor population, and a decrease in 5-HT signaling [24]. In healthy subjects, the G(-1019) allele has been associated with a predisposition to the development of a depressed phenotype, and panic disorder, or agoraphobia. Throughout the years, several rare single nucleotide polymorphisms have been also described for this receptors’ gene, for example, changes in the N-terminal domain of this receptor have been associated with altered desensitization (Gly22Ser mutant) or serotonin-induced response of the receptor (Ala50Val variant) [26,27]. Certain alleles have been also associated with a better response to the administered treatment. Thus, patients carrying s10042486 C/C and rs1364043 T/T mutants were believed to respond better to the antidepressant treatment [27]. Interestingly when it comes to the rs878567 mutant, its allele A is correlated to psychotic disorders in people suffering from substance-use disorders when exposed to methamphetamine. However, these data were established in the Japanese population, and up to this day, no association with other ethnic groups has been made [28].

Another important target that has been widely examined is the 5-HT_2A_ receptor (serotonin 2A receptor). Although numerous polymorphisms of serotonin receptors were identified throughout the years, two variants of the 5-HT_2A_ receptor gene have been correlated to depression symptoms. Thus, the presence of the 102T/C polymorphism and the AA genotype for the 1438A/G mutant is believed to be associated with the lower receptor expression and the occurrence of disease symptoms [28,29]. On the other hand, a large number of genetic studies failed to deliver information on associations between a certain genotype and the presence of the disease symptoms [30]. What has been confirmed is that there is a correlation between the structure of the HTR2A gene and response to the treatment with antidepressants [31,32]. Thus, the rs7997012 G/A polymorphism was correlated with a significantly higher antidepressant response rate in MDD patients [31]. Another gene-based association analysis chose 14 polymorphisms as the suggested markers for an antidepressant response, however, on further evaluation they did not reach statistical significance in the case-control study [32].

When it comes to other serotonin receptor subtypes, clinical studies data and information on their single nucleotide polymorphisms are not sufficient to correlate their features to the presence of depressive-like behavior.

Later, particular attention was placed on the protein named serotonin transporter (5-HTT, solute carrier family 6 member 4) encoded by the SLC6A4 gene. This protein is a common interaction point for numerous groups of antidepressants since their mechanism of action involves binding to SERT and reducing the serotonin reuptake. Among genetic differences which are believed to make an individual more sensitive to the development of depression, one can distinguish the following gene: biallelic 5-HTT gene-linked promoter region (5-HTTLPR), and its short allele which increases the susceptibility to depression [25]. Although the exact meaning of this alteration has not been yet established, HR girls homozygous for the S allele have been found to produce more prolonged levels of cortisol than the carriers of a second variant [26]. A single nucleotide polymorphism of A/G (rs25531) was also identified in proximity to the previously described region. In this context, the A/G substitution promotes the appearance of the L_G_ allele, which acts as a functional analog of the 5-HTTLPR S variant [27,28]. A few years later, another single nucleotide polymorphism was identified and comprised of a substitution C/T (rs25532). This combination changed the activity of the 5-HTTLPR/rs2551 polymorphism. In light of these findings, the combination of the L variant at the 5-HTTLPR polymorphism with the A/C substitution at the rs25531 and 25532 leads to a high level of the SLC6A4 expression [29]. Moreover, another study, that aimed to explore the relationship of the serotonin transporter gene genetic variants with the presence of depressive symptoms and elevated Interleukin-6 levels revealed that there are six genetic variants statistically important for the development of the disorder symptoms. These include rs8071667, rs2020936, rs25528, rs6354, rs11080122, and rs8076005 [30,31]. The identified relationship suggests that there may be a connection between common pathophysiological processes of depression and inflammation, and the serotonin pathway is an important component of such interactions.

One of the proteins which also influences the serotoninergic system is the brain-derived neurotrophic factor (BDNF). It is involved in a process of brain neuroplasticity, cell survival, and axonal growth. The Val66Met mutant of the BDNF gene reduces proBDNF carriers to the less frequent Met allele, which has been associated with a reduced capacity for managing stressful situations, anxiety, or impaired memory function. The debate on the exact meaning of this polymorphism remained a hot topic for more than a decade. First, it was reported that the A allele is more frequent in MDD patients, rather than in healthy control. However, more recently the presence of this allele has been associated with worse antidepressant outcomes, unfavorable clinical characteristics, and increased suicidal thoughts and anxiety [32].

Speaking of dopaminergic neurotransmission, the most widely examined target in this context is the dopamine D_2_ receptor, which is encoded by the DRD2 gene localized in chromosome 11. In a work published by Wang et al., a group of nine single nucleotide polymorphisms have been examined to identify their correlation to the response to the treatment with selective serotonin reuptake inhibitors (SSRI) and to explore the relationship between these SNPs and one’s susceptibility to depression. Three of the examined polymorphic variants named rs1076562, rs2440390, and rs2734833 showed significant association with MDD [33].

Moreover, many conducted studies also highlight the role of stress and the dysregulation of the hypothalamic–pituitary–adrenal (HPA) axis in the development of depression. It is possible to name a few single-gen polymorphisms potentially involved in this pathological process. The BclI and Asp363Ser mutations of the glucocorticoid receptor gene (GR), leading to hypersensitivity of this receptor, have been linked to elevated depression development risk in the general population [34]. The altered function of this protein may significantly disrupt feedback regulation of the HPA axis, and lead to its activation, which is an often observed phenomenon in depressed or chronically stressed patients. Moreover, some SNPs identified in proteins related to the GR heterocomplex activity regulation were also named as potential genetic factors changing the sensitivity of the glucocorticoid receptor. Substitution of cytosine by thymine in the FBPP5 gene results in the increased expression of the FKBP5 protein, which seems to negatively influence the hormone-binding affinity and translocation of the hormone-receptor complex [35]. Moreover, carriers of this mutation experienced slower recovery from a stress-induced cortisol response and therefore, such a feature may also be associated with having an increased vulnerability to the development of a depressive episode [36]. 

In 2020, Bai S et al. published an interesting systematic review, aiming to collect information on the effectiveness and safety of anti-inflammatory medication in the treatment of patients with the major depressive disorder [37]. The results of this analysis suggested that anti-inflammatory medication could exhibit an antidepressant effect in MDD patients, which stands for pharmacological proof supporting a valid role of inflammation in the development of this disease. Among other factors, genetics-derived data named numerous mutations impairing the functioning of, for example, interleukins or C-reactive protein (CRP), which could make patients potentially more vulnerable to the development of depression. In particular, interleukin-1-beta is considered a highly polymorphic protein. Mutations within its gene result in the increased severity of depressive symptoms, earlier onset age, and impaired response to some antidepressants [38]. CRP protein, on the other hand, is considered a systemic inflammation biomarker, which is commonly elevated in patients suffering from depression. A study published by Ancelin M-L et al. assessed depression in 990 elderly people and managed to genotype five SNPs in CRP protein-related genes potentially related to the development of mood disorder. Out of which two alleles, rs1130864 and rs1417938, were associated with a decreased depression rate in women [39].

Recently, attention was given to an exploration of certain alternative pathways, potentially involved in the pathomechanism of this condition. In particular, mitochondrial dysfunctions and impairment of cellular energetic metabolism have been linked to disruptions in neurogenesis and the development of certain mood disorders [40]. Moreover, animal-trial data confirmed that an increased expression of mitochondrial fission genes, along with a decreased expression of fusion genes are correlated with the presence of depressive behavior in rodents [41]. Genome-wide association studies have named numerous loci correlated to the elevated risk of disorder development, such as CACNA1C, SYNE1, or TRANK1 [42]. Studies of that type raise the possibility that the processes of energy generation and oxidative damage could be considered significant targets for the treatment of mood disorders. However, this path needs further exploration.

Although numerous studies have been constructed to identify the exact mechanism leading to the disorder, no conclusive information has been found. A growing quantity of evidence in this field and recent technological advances have enabled the application of certain genomic approaches, including next generation-sequencing, genome-wide association studies (GWAS), and candidate gene analysis in the determination of certain genes involved in the pathological process. Thanks to that it was possible to link one of the disorder’s phenotypes with a certain genetic representation and learn more about the genetic architecture of this disorder [41,42,43]. In the past decade, we have witnessed a significant increase in the efficiency of these studies. With the continuous growth of sample size, it was possible to increase the number of returned meaningful correlations from 2 to almost 180 loci [44].

Although, genome-wide association studies have been widely applied for the discovery of new candidate loci involved in the characteristic molecular “fingerprint” of depression, currently, epigenetic factors are starting to gain a lot of scientific interest [45]. The process of methylation of DNA occurs due to the transfer of the methyl group of S-adenosyl methionine and leads to the formation of carbon-5 cytosines. It is considered a heritable and reversible modification. Moreover, it is catalyzed by DNA methyltransferases, which establish and preserve characteristic DNA methylation signatures in various cells [45,46]. This process is believed to regulate gene expression within the Central Nervous System; thus, wrong methylation patterns are believed to result in the development of certain neuropsychiatric disorders. In a very thorough review published by Shirvani-Farsani et al., an overview of recent studies aiming to identify a correlation between the presence of, e.g., major depressive disorder and a certain methylation pattern genotype has been presented. Among all these results, certain genes such as, for example, ID3, GRIN1, and TPP have been correlated to methylation changes in children. Surprisingly, the higher prevalence of the methylation of NR3C1 promoter in infants was noted in the children whose mothers suffered from postnatal depression, which was followed by relatively low prenatal depression. Moreover, in women who did not have prenatal depression but developed postpartum depression, an interaction between rs53576 and the methylation of exon 1 has been established. This genotype decreased DNA methylation in female patients [46,47,48]. Unfortunately, the enthusiasm for epigenetics is still limited by the small range of described changes, especially in comparison with other conditions. 

## 4. Genes Linking Depression with Other Disorders?

Comorbidity (co-existence of two or more mental disorders in one individual) is a relatively common phenomenon, with prevalence estimated for more than 40% of cases [49,50]. In the case of major depression, a patient has a 10–20% risk of developing a second disorder. The prevalence of developing these symptoms is even higher in people older than 65. Throughout the years, numerous studies have been conducted to identify the risk for patients to develop depression as a co-existing disorder.

One explanation for this association is the fact that both conditions share a common genetical basis. Therefore, shared genetic vulnerability makes a patient more sensitive to developing two distinct disorders [51]. Another theorem refers to external pharmacological factors. Thus, chronic administration of certain therapeutics may result in the presence of mood-diminishing side effects. These symptoms are most widely recognized in patients submitted to certain groups of medication, e.g., beta-blockers, calcium channel blockers, antiobesity drugs, and progesterone [52]. Despite the prevalence of this theorem in scientific journals, vigorous evidence for such an association is lacking. Moreover, scientific literature mentions more factors that could be involved in this phenomenon, and those are: unhealthy lifestyle, lack of adherence to medical treatment, and psychological factors [53]. Triggering factors are as yet poorly understood, however, what seems to be more interesting in this case, is the most commonly observed disease combinations.

There is a large body of evidence confirming a high interconnection of depressive disorder with other mental disorders. It is estimated that approximately 25% of general practice patients with MDD will additionally develop an anxiety disorder [54]. Both disorders are identified with a 2:1 ratio for women and men, respectively. A recent neurological meta-analysis confirmed common alterations in the salience, reward, and lateral orbital non-reward networks, which contribute to a similar pathomechanism theory [55]. Moreover, data coming from transcranial magnetic stimulation studies imply that targeting different regions within the left dorsolateral prefrontal cortex result in a reduction in certain symptoms [55,56]. Other psychiatric disorders which can co-exist with depression include schizophrenia, substance drug abuse, bipolar disorder, post-traumatic stress disorder, and obsessive-compulsive disorder [57,58]. These combinations are usually harder to treat since they involve a cocktail of medications to receive a proper therapeutical response from a patient. Moreover, the co-existence of two or more psychiatric disorders increases the risk of sociological exclusion of a patient and elevates the mortality rate [59,60]. Unfortunately, criteria for the identification of co-existing psychiatric disorders are not selective enough, because they are mostly based on the clinical picture of the disorder, and include numerous common symptoms for many disorders.

**Figure 3:**
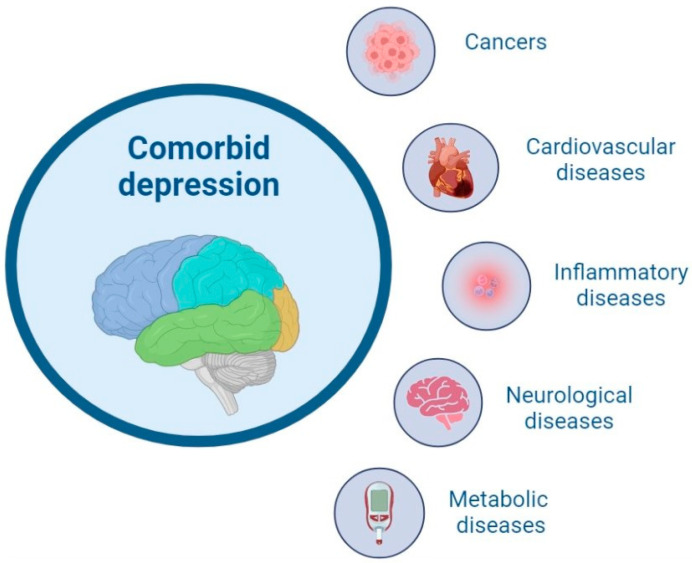
The most common groups of diseases that co-exist in patients with depression [6].

It is not surprising that the co-prevalence rate of depression with other physical disorders varies (Figure 3). Approximately 16% of patients diagnosed with cancer seem to develop depressive symptoms. Although it is hard to compare studies published in this field, patients suffering from ovarian cancer and brain tumors seem to be the most vulnerable [53]. Moreover, 31% of stroke survivors are expected to develop depression in their later life. On the other hand, approximately 28% of patients with myocardial infarction suffer from comorbid MDD. However, the disease occurs more commonly in women than men [61,62]. Other cardiovascular diseases that seem to frequently co-exist with depressive symptoms include heart failure and peripheral artery disease. It is worth emphasizing that the recent data suggest a two- or three-fold higher depression prevalence ratio for individuals with other physical diseases [60]. An extensive review published by Gold et al. also mentioned a high prevalence of co-existing MDD among people diagnosed with diseases of metabolic syndrome (e.g., 23% of men, 34% of women with diabetes type 2) and inflammatory diseases (e.g., psoriasis, rheumatoid arthritis) [53]. Factors believed to make a person more vulnerable to the development of two or more co-existing conditions are associated with a person’s genetic predispositions, environmental factors, and lifestyle.

In addition to a relatively high prevalence of comorbidity in depression, it is worth emphasizing that such a condition complicates patients’ overall disease management and treatment plan.

## 5. Approaches Promising for Treatment-Resistant Patients

As was emphasized throughout this manuscript, depression is a disorder of numerous phenotypes, and it is practically impossible to name one cause leading to its formation. Therefore, designing medication capable of equally diminishing disease symptoms in all depressed patients is considered to be a massive therapeutical challenge. Even though numerous groups of so-called antidepressants are currently marketed and available for both doctors and their patients, these medications participate in the onset of numerous side effects, diminish only symptoms of the disorder, and do not cure it.

Data coming from the clinical investigation revealed that treatment resistance is a problem of serious concern. This phenomenon can be identified in 42–53% of patients suffering from major depressive disorder [63]. Thus, much more attention should be paid to the exploration of approaches capable of optimizing current therapeutical strategies. In this subchapter, numerous relatively promising approaches for the improvement of the therapeutical effectiveness of administered medication will be discussed.

Currently, many potentially effective strategies for the maintenance of treatment-resistant forms of major depressive disorder have been developed. Those include:

- Augmentative or adjunctive therapy (using a second medication, which in most cases does not belong to the antidepressant category, e.g., including a lithium-containing agent as an addition to the standard tricyclic antidepressant pharmacotherapy);
- Optimizing, combining, or switching classes of administered antidepressant medication;
- Psychotherapy in addition to some somatic or pharmacological treatments;
- Different forms of brain stimulation therapy were proven to be successful as an alternative therapeutical strategy [64].

Moreover, recent advances in pharmacogenomics make it a promising tool for combating treatment resistance in certain individuals. In some patients, the presence of specific genetic variants of CYP2C19 and CYP2D6 makes it harder to reach the level of optimal exposure to antipsychotics or antidepressants [65]. The abovementioned proteins belong to highly polygenic transport enzymes of the cytochrome P450 superfamily. Different alleles encode the phenotype of extensive, poor, intermediate, or ultra-rapid metabolizers, that are capable of metabolizing the enzyme substrates at different speeds [66]. Therefore, implementation of a pre-emptive CYP genotyping could help determine the patients’ metabolic genotype and properly adjust the dose of medication [63]. A meta-analysis conducted by F. Milosavljevic et al. concluded relevant 2.6- and 2.7-fold differences in escitalopram and sertraline exposure between different groups of CYP2D6 metabolizers [67]. Moreover, large-population studies aiming to determine the advantages of pharmacogenomic tools for pharmacotherapy optimization noted significant improvements in symptoms in a group of CYP-guided MDD patients [68,69,70]. Thus, implementing routine pharmacogenomic testing could offer important clues in drug selection and dosing, especially for people having a previously documented poor therapeutical response to commonly prescribed medication. Up to this date, several companies have marketed commercial combinatorial pharmacogenetic tests for such purposes (e.g., “The AmpliChip CYP450” test—Roche, Basel, Switzerland). These tools take into account a limited number of genetic variants, therefore, it is possible to obtain reliable information with lower costs and computational time.

Polymorphism studies also target other, potentially important disease pathomechanism proteins. Heretofore, numerous studies aimed at collecting the resources available in this field. In a meta-analysis published by Porcelli et al., a special emphasis was put on the data targeting serotonin transporter protein (SERT), which is considered one of the main targets for antidepressant medication and a primary target for compounds from the SSRI family. The main aim of this manuscript was to try to correlate polymorphism in the serotonin transporter gene promoter (5-HTTLPR) with the expected response to antidepressants. Collected data suggested that for the Caucasian ethnic group, this parameter could be considered a predictor of antidepressant response and remission, whereas in Asians there is no significant proof of such a correlation [71]. Moreover, a few other drug–gene interactions have been described in the literature, e.g., *B-1502 allele of human leukocyte antigen (HLA) which is associated with carbamazepine-induced Stevens–Johnson syndrome among the Chinese population [72]. In numerous studies, single-nucleotide polymorphisms in genes encoding 5-HT_2A_ and 5-HT_1A_ receptors were proven not to be significantly correlated to the individual’s response to the SSRI treatment [73,74,75,76]. Additionally, in terms of pharmacotherapy optimization, it is important to take advantage of deposited external resources and pharmacogenomics data. One example of such a server is the Pharmacogenomics Knowledge Implementation server (PharmGKB), which collects evidence for gene–medication interactions. Collected data refer to significant associations with strong effect size replicable in multiple cohorts, as well as data collected during preliminary studies [76]. One of the most popular selective serotonin reuptake inhibitors, which is commonly used in clinical practice, is sertraline. Data samples on significant pharmacogenomics aspects, referring to the efficacy of this compound, collected from PharmGKB, are presented in Table 1.

Although up to the current date, no spectacular data have been retrieved from genome-wide analyses, it is worth emphasizing that most of the currently published studies were based on a small population sample. Thus, expanding the scope of analyzed data could benefit the results and provide some strong evidence of desired correlations. The translational part of pharmacogenomics is not available in the doctor’s office yet. However, with multiple estimates taken into the account, this evidence can provide valuable insight into the pathomechanism of the disorder, the complexity of the human organism, and the pharmacological action of commonly prescribed antidepressants. 

The constant need to develop more effective therapeutical strategies results in the identification of large amounts of information referring to the novel molecular targets expected to be involved in the pathomechanism of this disorder. Recent findings suggest that ligands of opioid receptors could be a potential hit for the medication of the treatment-resistant form of the disorder. Numerous studies have been published evaluating the effectiveness of buprenorphine, tianeptine, and other ligands in this field. Opioid-receptor targeting strategies turned out to be effective for patients with treatment-resistant depression, which could lead to the conclusion that the exploration of molecular targets other than monoamine receptors is a promising direction [79,80,81,82,83,84,85,86]. On the other hand, exposure to N-methyl-D-aspartate receptor antagonists such as ketamine, nitrous oxide, memantine, or lanicemine resulted in a reduction in depressive-like symptoms in treated MDD organisms [86,87,88]. Other targets which are considered to be a promising direction in antidepressant-oriented drug campaigns include, for example, peroxisome proliferator-activated receptors (PPARs), G-Protein Coupled Receptor-39, metabotropic glutamate receptors, galanin receptors, etc. [80,81].

The search for new medication capable of diminishing disease symptoms in patients with MDD is a project that involves all branches of medicinal chemistry. No wonder that in the literature we can also find information about drug repurposing campaigns, aimed at finding new effective agents among already marketed compounds. Approaches such as, e.g., signature matching, molecular docking, or previously described Genome-Wide Association Studies (GWAS) constitute the computational core of drug repurposing campaigns, while retrospective clinical analysis, binding assays, and phenotypic screening are commonly applied for experimental campaigns [89,90]. These tools help to decrease both expenses and the duration of the drug discovery campaign. To date, numerous repurposed therapeutic agents have been approved by the US FDA, e.g., aripiprazole, buxanolone, and esketamine. These attempts shed another light of hope among patients, suffering from MDD, seeking the therapy capable of restoring their comfort in life [91,92,93].

## 6. Conclusions

According to current epidemiological data, depression is a disease of serious concern with a noticeable impact on society. Although the exact prevalence of this disorder varies depending on region and country, overall it is considered one of the leading causes of disability worldwide. Patients carrying genetic, environmental, and pharmacological vulnerability factors, go through the first onset of the disorder mostly triggered by a serious emotional experience.

Despite the over-the-year-long research on the pathomechanism of the disorder, the exact mechanism leading to the development of symptoms has not been established. Even though depression certainly has a heritable component, it remains challenging to elucidate with the current sample size, mostly due to its polygenic nature. Fortunately, with the help of pharmacogenomics, we are capable of enumerating genes correlated to these pathological changes and interpreting these data to establish a better understanding of the disease phenotype and the mechanisms underlying the very common lack of therapeutical effectiveness of the typically administered medication. Implementation of rapid genetic profiling as a part of a depression assessment could help to adjust the treatment to the individual needs of a patient. From a long-term perspective, it would benefit adherence, possibly reduce the severity of observed adverse reactions, and lower the treatment expenses. 

The complex nature of the disorder and numerous genetic associations make a depressed patient more vulnerable to the development of other conditions. Comorbidity may also result from an adverse reaction to a medication. Regardless of what mechanism leads to the formation of these co-existing conditions, it elevates the diagnostic challenge, since the current diagnostic criteria do not fully reflect subtle differences between conditions. Moreover, it requires special therapeutical adjustments and precautions to eliminate drug–drug interaction risk. In the case of depression, comorbidity significantly increases the mortality rate, thus, this group of patients should be submitted to special care. 

The special impact that depression has made on society has been noticed by scientists. Numerous attempts have been made to establish more data on this pathology and create novel, more potent medications. Since currently available medications are of varying effectiveness, it is necessary to explore alternative directions and test compounds with novel mechanisms of action. Such approaches include targeting opioid or 5-HT_2A_ receptors. Unfortunately, the drug discovery pipeline is a long process, and drug repurposing campaigns are being conducted to identify novel antidepressants, which could rapidly improve implemented therapeutical strategies. In addition, fast genetic profiling seems to be an interesting approach, since it would enable the adjustment of the medication scheme to an individual patient’s needs.

## Figures and Tables

**Table 1:** Sertraline—popular selective serotonin reuptake inhibitor along with significant pharmacogenomics data, referring to the efficacy of this compound.

Substance Name	Gene (Variant)	Association
SERTRALINE	GNB3 (rs5441) G- protein subunit beta-3 gene	Patients holding a GG genotype were significantly more likely to respond to sertraline than those with AA or AG genotypes [77].
	ACE (rs1799752) Angiotensin I converting enzyme	Patients with depressive disorder holding del/del genotype are associated with an increased response to sertraline [78].
	SLC6A4 (SLC6A4 HTTLPR long—l allele; short—s allele) Solute carrier family 6 member 4	SLC6A4 HTTLPR short form is associated with an increased response to sertraline in people with major depressive disorder (as compared with l-allele) [79].

## Data Availability

Not applicable.
